# Supplementary material for: Experiencing the COVID-19 pandemic as a homeless person in Chennai, India: An interpretative phenomenological analysis
Source: PLoS One. 2023 Nov 30;18(11):e0295164. doi: 10.1371/journal.pone.0295164 (PMC10688851; doi:10.1371/journal.pone.0295164)
Supplement: S1 File — (DOCX) [file pone.0295164.s001.docx]

**Experiencing the COVID-19 Pandemic as a Homeless Person in Chennai, India: An Interpretative Phenomenological Analysis**

**S1 Prompting Questions:** The list of key and ancillary prompting questions used in the semi-structured interviews

1. what was your opinion on COVID-19 when you first heard about it?
   1. When news about the virus in China started spreading, how did you feel about it? Did it seem like a distant thing or did it hit close to home?
   2. What was your gut reaction?
2. How did the pandemic affect you?
   1. How did the pandemic change your everyday life? What was different from before?
   2. What adjustments did you have to make with work, where you live, and how you interact with others?
   3. How did it feel to not see friends and family? Did you ever feel alone or disconnected?
   4. How did you manage to stay in touch with your loved ones when you couldn't really see them in person?
3. How have you been coping with the situation?
   1. Any survival tricks or activities that kept you sane?
   2. Anything that helps you deal with the tough times, like any small joys or distractions that help?
   3. Did you see things from a different angle due to all of this?
   4. When things were tough, did you find any meaning or lessons in the situation, even though it was hard?
4. Have you needed a doctor since the pandemic began?
   1. How did you go about getting it?
   2. Did your worries about getting sick change over time? Did you feel differently about it as things went on?
   3. How did you handle concerns about your health and your family's health?
   4. What were your thoughts about healthcare during this time? Did it change how you think about doctors and hospitals?
5. What are you looking forward to once things start getting back to normal?
   1. How do you think life might change as this whole situation evolves?
   2. Any plans? What are you hoping for?
