# Supplementary material for: Experiencing the COVID-19 pandemic as a homeless person in Chennai, India: An interpretative phenomenological analysis
Source: PLoS One. 2023 Nov 30;18(11):e0295164. doi: 10.1371/journal.pone.0295164 (PMC10688851; doi:10.1371/journal.pone.0295164)
Supplement: S2 File — (DOCX) [file pone.0295164.s002.docx]

**Experiencing the COVID-19 Pandemic as a Homeless Person in Chennai, India: An Interpretative Phenomenological Analysis**

**S2 Appendix:** Translations of all noteworthy utterances of each participant that underpinned the analysis

*Participant identification number (PIN)1*:

“I had seen the news. They said there is some new virus in China and it is infecting a lot of people and many are dying. I felt that somewhere in China if people are getting infected, why should we in India be so scared? The government is unnecessarily putting us all in a bad situation. We should just go about our lives, it is not our problem”.

“The lockdown felt alright at the beginning. I was getting food and I just had to relax. But then we are not living just for food and shelter, we have many other needs. I have no work. I sleep on the street so that I can send money to my daughter. She needs it to study. She is a very good student. I want to make her a doctor. But I have not sent any money home since the lockdown. Nothing is open, where will I find work? My daughter must pay tuition fees soon, or the tuition master (private tuition) will not let her attend classes. Corona does not even seem to end; I don’t know when I can work and send money to my daughter again. If she fails because she was thrown out of tuition, I will never be able to forgive myself”.

“My parents are old. I heard that old people cannot survive Corona. I am worried about them. Even after travel has been allowed, I decided not to go back home because I don’t want to take the infection with me and give it to them. My father does not like sitting at home, I am so afraid that he will go out and get infected. But what can I do? He won’t listen to me or anyone else so there is no point in trying. I just hope that he is seeing all the news and stays in the house. There is so much death everywhere and everyone is scared. I don’t know about everyone but at least I am scared and it is all the rich peoples’ fault”.

“The rich people knew they will bring Corona to India if they travel now, but still they travelled. Not just that, they lied to the government about their symptoms and spread the disease to innocent poor people who drive their cars, and work in their houses. Then they happily took the best medical care and left the poor people to fend for themselves. Suppose Corona comes, the rich will immediately get beds in big private hospitals but people like me have to wait for someone to see us in public hospitals”.

“Private hospitals are for the rich; people who live a hand-to-mouth existence like me cannot even think of them. They will gobble up the five lakhs insurance money in one day and ask us to get out the next day. It is coming in the news, didn’t you see? They have increased the prices so much that even if you go to see them now with tooth pain, they will make you sell some body parts to foot the bill. They are making money on dead bodies”.

“I videocall my family over WhatsApp and talk to them for at least an hour every day. It is the bright spot in my day. Whenever I get bored, I watch videos on YouTube. There are so many interesting things to watch there. Then there is news, which is streamed live on YouTube by various regional and national news channels. When I am watching videos, I don’t know how the time passes”.

*PIN2*:

“Initially I didn’t think it was such a big thing. Just another fever”.

“So many people are dying. There is so much fear in everyone. I am also scared. I am the only earning member of my family. If something happens to me, my family will have nobody to support them. The place I go to get my food is always crowded; I am so scared to get my food that I have even thought of skipping meals”.

“I have thought about skipping meals, but hunger is hunger. Thanks to God, I have not gone a single day without food. Before Corona, sometimes I would get only one meal per day, but now, I get three meals daily from an NGO. But how long will I live on charity? I offered to wash their utensils, during lockdown, they refused. But recently they agreed. Now I can get meals and don’t even feel like I am getting it for free”.

“We are having a lot of fights in the family because of this lockdown. My father is frustrated about being confined to the house, and whenever we talk, he takes it out on me. He is always picking fights with me over every small thing. I am also very much frustrated with the situation. But obviously I can’t argue with my father, so I show my irritation to my mother. But you know what? Fighting is not a bad thing for a family, it shows we love each other, no? If there is no love, will there be anger? Other than this I can’t think of anything else that stressed me during the lockdown”

“The restrictions are being lifted and I was hopeful of going back to work. But my employer told me not to come back. She is afraid I might give her family Corona. She does not want anyone from outside going to her house. Especially me, because she says I don’t have a house so it will be impossible for me to take all safety measures. She told me she is afraid of letting me in the house. I felt bad but what can I do? It is her house, she is paying me, I can’t say no you have to let me come in and you have to pay me, right? For now, the food is there, soon I hope I will find work again. Maybe my employer will call me back or I will get a new house to work in. I have been tested multiple times now. The Corporation (local government) officials took my phone number and regularly call me to find out if I am having any symptoms and if I need any help. Not once did I feel judged. Because I don’t have to worry about paying for it, I go for testing whenever I get any symptoms. I just hope to show my negative results and maybe even promise to get tested regularly and someone will give me work”.

*PIN3*:

“I didn’t think much of it...I thought it had nothing to do with me! But look now! The news, Facebook, everywhere you see, people are only talking about how many people are getting infected in the city. The threat of Corona has become very real”.

“I have not gone home for so many years, but I always thought I was only one train journey away. But they have stopped the trains now, and I am stuck here. I cannot meet anyone, nobody will come to meet me, and I have not spent time with another human being ever since this disease started spreading. All this is because of these rich travellers…I have seen the news how they took medicines to lower their fever just before coming out of the planes to escape the authorities. Some of them escaped from the quarantine facilities. What will happen if they spend 14 days in government quarantine? Will they die? They lied and spread the virus everywhere. These rich people are responsible for every single Corona death in this country”.

“I am not afraid of Corona. I am strong, so I will not get affected by it. But the Prime Minister said on TV that even if we don’t get infected, we can give the infection to others. I am always conscious of how close I am to another person. I am worried if someone comes too close to me, what if I get Corona? I will not get affected, but that means I will not know I have it. Then if I touch someone else, and they get it and they die? I will never be able to forgive myself. Ever since this Corona started, I have not touched another human being. I crave for the human touch, it makes me very sad to not touch another person, but I am worried I might spread Corona to them. I don’t want anyone to suffer because of me. My wife is constantly nagging me to come home. I still have a job, if I go home now, I will not have a job to come back to. My wife refuses to understand this, she just wants me to come home. I already miss my family; will I not go home if I could? And what about the risk of spreading the virus if I go home? I am tired of explaining all this. It is causing me a lot of mental strain”.

“I work in the nights. In the morning I sleep. When I am awake, I message my friends and see their photos on Facebook. Sometimes I call them as well. My wife calls me on WhatsApp, and I see my parents and my son on video. I am happy to see them like this; they can be safe in the village and I also get to spend time with them. Technology has made it easier to tide over this crisis”.

*PIN4*:

“Nobody has heard about Corona till the Prime Minister came and asked us to participate in Janata Curfew. Even at that time, my family, my friends and everyone I knew thought this is not something we should worry about. People who travel to China have to worry about it. Now, after so much death, thanks to these people who came here from China, we all are scared of this Corona. I am healthy and I want to stay that way. I started washing my hands with soap after every three hours. I try not to meet anyone unless it is necessary”.

“When will this Corona leave us? Will we ever get our life back? Only God knows. How can I make plans without knowing what tomorrow will look like? This uncertainty makes me very anxious. And there is not that much construction work going on now. But I thought I will do any work that I can find. But I am not able to find anything. I tried for a job at a small eatery, but the owner threw me out. He said I am shabby and if he hires someone who looks like he sleeps on the road, whatever customers he might get will also run away, because who knows what germs I will bring with me. It was very hurtful, but such is life, what can be done”.

“I don’t like going to private hospitals. The hospital staff are okay, but the patients and their families look at people like me as if we are dirty. If I ever need to go to a hospital, I will wait till Corona is no longer a problem, and then go to a public hospital. I can only afford public hospitals, but they are crowded. Everyone keeps saying that we must avoid crowds. Why go to the hospital and risk getting Corona...chest pain is more likely to be just gas. The odds that I am going to have a heart attack at my age are slim. But the odds that I will get Corona if I go to the hospital are high”.

*PIN5*:

“At that time, I thought, if you go to China, you will get it. Where do I have the money for that? I don’t have money to go to my village itself. Rich people who travel here and there should be worried, not people like me”.

“I ran away from home because I was a burden to my family. I came to the city and did some odd jobs. Whatever job I got, I did it wholeheartedly and ate whatever I could afford with my own money. I even started sending small sums of money to my family. I was fine with even sleeping on the streets, but I wanted to stand on my own feet. But ever since this Corona happened, I have not had any work. I am completely dependent on some kind hearted persons to provide me food. I hate my situation. I have to do what I ran away from home and slept on the streets to avoid doing; I am eating free food”.

“Everything I have strived for has gone to waste. Whatever principles I have stood by and even endured the humiliation of living on the streets for, I have now violated. There is no meaning to my life now”.

“I have joined a prayer group on Facebook. Every evening, I light a lamp in front of God, and open Facebook. The group admins do live streaming of Bhajans. I forget all my troubles and sing along with them for one hour. It is the only time I feel that my life is not a waste”.

“I used the Ayushman Bharat scheme at a famous private hospital. They treated my condition, but as long as I was there, they never looked at me like just another human being. I was always the guy who lived on the street. Nobody likes being looked at like that. I never want to go to any private hospital anymore. I will try to just stay healthy or if I need to, I will go to the Corporation clinic or the public hospital. Especially in these Corona times, I am seeing the news about the private hospital bills. So much looting is going on…better to take care of your health than to fall prey to private hospitals. And if God deems that you need to see a doctor, just go to the public hospital”.

*PIN6*:

“When we had the Janata Curfew, I heard of Corona for the first time. But now I think why did I have to hear that word…life has been tough since the day I knew that word. Not just for me but for everyone around me. If people just had little social responsibility and did not travel back from China, we would not have this disease. Then all people who left the country for money started coming back, bringing Corona from those places even though we closed our border and tried to stop Corona from coming. The Government should have been a little more strict and told these people to just stay where they are…they should have closed the borders for these people also, not just foreigners”.

“When I was a kid, my father taught me Yoga. I practice it every day to keep healthy. Especially since this lockdown started, I have not missed practice even a single day. I practice on the footpath where I sleep. One day a Policeman saw me practicing. He asked me if I wanted to become a Yoga teacher. I told him I would love to, but I don’t have money, and this is the life I am destined for. The Policeman asked me if I have a smart phone. I told him yes. He took my phone and enrolled me in an online Yoga teacher training program. He paid for it. The program is in English, but the teacher repeats everything for me in Hindi. Every time he sees me, he asks me how I am doing in the program. I will be a certified Yoga teacher soon, and I will be able to find a stable job and move out of the streets. I don’t have to live this humiliating life forever, all thanks to that man’s kindness which I can never repay in my life”.

“There are two other people where I sleep, we usually eat together and talk to each other when we come back to our corner after the day’s work. I thought they were my family away from my real family. But when the government lifted the lockdown, they have gone back to their families, and I am left with nobody”.

“I have been praying since I was 12 years old. I have not missed a single Friday prayer, but lately, I have been thinking, if God is inflicting so much suffering on innocent people, is he really fair? I don’t feel like praying anymore. I just cope by doing Yoga. At the beginning, the Prime Minister used to come on TV and motivate us. I used to look forward to the next task he will give the country. When I clapped for the doctors, lit a candle, and did not venture out during the Janata curfew, I felt collectively we will beat Corona. First time in many years, I felt like a part of this country, a part of a good cause. But now, the health minister comes on TV. He says something and goes. I do not understand much. Slowly that sense of belonging to a cause has vanished. I hope the Prime Minister comes back to motivate us again. It will help our people to cope with these times”.

*PIN7*:

“I didn’t know anything about Corona before lockdown was announced. When the lockdown was announced, I was washing the dishes…my employer asked me if I wanted to stay in her house and continue to work instead of going home and losing out on the money. I said I will stay. I thought I will have work, and have company to deal with the situation. But I felt lonely. They gave me food, shelter, and my wages. But they were not my friends or family. They can never understand what I am going through because they have never been in my situation”.

“In the place where I work, I have to get the food items for the employers. Another housemaid I know got Corona by going to the market for food, and after getting Corona, she passed away. If the same happens to me, who will take care of my family? I try to keep my distance from others there, but I am scared for my safety. I am constantly worried about getting infected. I don’t want anyone to come near me. I have stopped taking my wages in cash. What if the virus is on the notes and I get infected because I used cash? I have started using BHIM to get my wages and to pay for anything. When my employer asked me to buy things for her house every day, I told her to get gloves for me. When I go to the shops, I don’t go without the gloves and once I deliver the items, I throw away the gloves. How else can I be sure that I will not get Corona from the things I touch at the shops? If someone coughs or sneezes at the shops, I run away and go to another shop. I cannot afford to get infected. Not just Corona, I don’t want to get any disease at this time especially”.

“Corona can kill me, but any other smaller thing is also a risk and a waste of time…if I go to a public hospital, I will have to be there the whole day, because the queue to see the doctors, and for tests, and everything else is so long. I don’t have that kind of time; I have to work. Besides, the longer I stay outside, the higher my chances of getting infected with Corona; I don’t want to take that chance”.

“I have only prayed for three years during my life, when I was living with my parents. I believe in God, but just did not see the point in praying. But ever since Corona started, I have been praying every day. I pray for the virus to be destroyed and our lives to go back to normal again. I saw a video where the Maulvi said that if we all prayed together, the virus stands no chance. I thought why not give it a chance. Once I started praying, it gave me a lot of peace. I pray every day now”.

*PIN8*:

“Have you seen where I sleep? There is so much filth. Everyone says that you should be clean and keep the surrounding clean or you will get diseases. But in so much filth, I am very healthy. I have not missed even one day’s work in so many years. My body can withstand anything. This corona is not going to harm me. Rich people who are worried about falling sick because of the rain should worry. They only travel to other countries and bring these diseases here and only they become sick because their bodies are not used to microbes. But government has imposed lockdown and we all have to suffer because of them”.

“I started going to work once the restrictions started easing. But nobody at the office comes near me. Nobody eats with me. Whenever I must touch anything, I must show the office staff that I am using sanitizer, only after that I am allowed to touch it. When I asked my supervisor why I must sanitize my hands so many times, but others peons don’t have to, they said it was because I live on the street; it is not likely that I will maintain hygiene and social distancing on the street. I may be immune to Corona because of my street-dwelling, but others are not like me. It is a great thing that I got a job at all, and it is an even bigger thing that after Corona and the lockdown, I got to keep my job, so I don’t complain much. But inside I get irritated and I always think…our country did not have this disease; it came from outside and the rich brought it here. They spread the disease and now they are acting as if we poor will give them the disease and want to be as far away from us as possible. If anything, we should be treating them like untouchables, not the other way around”.

“I know someone who also lives on the street who tested positive for Corona. The Corporation sent an ambulance to pick him up. He said he was taken care of very well; he was given three hot meals a day, and juice, tea, and coffee whenever he wanted. He had a clean bed, and he was treated for free. Ever since I came to know about the amenities at the quarantine facility, I have been thinking that getting infected with Corona may not be too bad; I can food and a decent bed to sleep on. But what if I get a severe attack, so I take all precautions and avoid getting infected”.

“There was nothing else to do, so I started learning English on Youtube. When I go to get my food, I talk whatever I learned that day with the food distribution volunteers. They laugh at my mistakes, but they also correct me when I am wrong and help me practice small conversations. If I can talk English, I will be able to find a better job. I am looking forward to such a day”.

“I don’t think there is a better method to forget your troubles and sleep peacefully than daily sex. My wife is in the village, and we must maintain distance from strangers, so I masturbate every night, once it is dark enough and I am sure nobody can see me. It calms my nerves”.

*PIN9*:

“I thought it is China’s problem, but it has become the world’s problem…we have to accept the reality. Corona is not going away just because we wish it to go away. I have to wear a mask and if I get a fever I have to go to the (Municipal) Corporation health centre and get tested”.

“They took what was China’s problem and made it the world’s problem. Why did they have to travel to China when news of the virus came? And if they went, why did they come back even after knowing about the virus? These people can’t live without luxuries even if it means poor people will die because of them”.

“I have to eat whatever other people decide to give me. I don’t like rice, but they distribute only rice packets every day. If I say I don’t want rice, give me roti, I will be ungrateful. I miss making my own food, but I have no money. I sent whatever money I had to my family, thinking that they are in the village and it will be difficult for them to find work there, and the lockdown was only supposed to be a matter of two weeks. I thought I can survive on whatever the government gives for the two weeks and then earn again. But I was wrong. There has been no work for months now. I am still surviving on doles. It is very frustrating”.

“I don’t have a roof over my head. I was sending all my money home so that they can have a better life, but now I have no money to send. If this continues, soon my family will also have to join me on the streets. I have no opportunities. I used to hope for a better tomorrow. Now I only pray that tomorrow is no worse than today. But I am coping…life has to go on”.

“I have always used opium and tobacco. They take my mind away from my reality and keep my calm, even happy. But now I use them a lot. I have not tried anything new; I think it is better to stick to what your body is used to. And I like these tasks that the Prime Minister gives on TV. I was very reluctant to do all those things that he asked the people to do on TV. But I started clapping for the doctors just like he said, because everyone around me was doing it and I did not want to be the odd one. But as I clapped, I became more enthusiastic. I felt like a part of something big and important. Then when he came on TV again, I looked forward to his task. My job loss and not knowing what will happen tomorrow seemed to be in the interest of this important cause of beating Corona. I felt like a soldier, fighting for myself, my loved ones, and countrymen. It has been a great motivator and source of strength”.
