# Supplementary material for: Experiencing the COVID-19 pandemic as a homeless person in Chennai, India: An interpretative phenomenological analysis
Source: PLoS One. 2023 Nov 30;18(11):e0295164. doi: 10.1371/journal.pone.0295164 (PMC10688851; doi:10.1371/journal.pone.0295164)
Supplement: Attachment — Submitted filename: plos article comments.docx [file pone.0295164.s003.docx]

Line 71 i.e. the operational definition of houselessness should be put up explicitly. The consenting procedures should be specified well ie verbal or written, and mode of language. Methods should have more clarity as to how PEH was approached ie through a civic or political body to gain their trust and confidence. Due to the rarity of these studies, it would be good if the consenting format may be a part of the submission (additional files). The ice-breaking sessions and conclusion of the interview i.e. the rights of these subjects in terms of time and sharing their experiences should be described as, were they offered free check-ups/food or monetary compensation for their narratives, should be described in better detail.

Line 603 all PEH in the study having the Ayushman Bharat card is a big limitation. Hence policy implications are limited as the benefit far outweighs the risk. The riskier are those who did not have the card.

Sociodemographic profile like education, family history, and reasons for living alone should be probed further. The impact of Covid 19 is looking better for this population due to heightened government activities like free food etc, so post covid restrictions pins would describe their situation better.

The age group is all in the sexually active period and given the conditions, it’s strange that they choose to stay alone. Chennai is the hub of AIDS & HIV, more details about their addictions and sexual habits would have added merit to the study.

A word on Covid 19 vaccination for this group would also be useful.

Study is good but the limitations are many and are to be discussed in more detail for benefit of readers. It qualifies to be a review article.
