# Supplementary material for: Experiencing the COVID-19 pandemic as a homeless person in Chennai, India: An interpretative phenomenological analysis
Source: PLoS One. 2023 Nov 30;18(11):e0295164. doi: 10.1371/journal.pone.0295164 (PMC10688851; doi:10.1371/journal.pone.0295164)
Supplement: Attachment — Submitted filename: Review PEH 2.docx [file pone.0295164.s004.docx]

Line 56

In India, 41.6% of PEHs have no access to healthcare.10 (modify this as Ayushman Bharat since 2018 is constituted to help these people and you have also taken up such people)

Line 89

purposive homogeneous sampling, (what was homogenous here) , you have gender stratification , so lets put it as purposive sampling

line 666

one cannot anticipate no difference, not taking those without AC can be stated as a frank limitation
