# Supplementary material for: Experiencing the COVID-19 pandemic as a homeless person in Chennai, India: An interpretative phenomenological analysis
Source: PLoS One. 2023 Nov 30;18(11):e0295164. doi: 10.1371/journal.pone.0295164 (PMC10688851; doi:10.1371/journal.pone.0295164)
Supplement: Attachment — Submitted filename: Response to Reviewers.docx [file pone.0295164.s005.docx]

Point-by-point response to reviewers

Line 56

In India, 41.6% of PEHs have no access to healthcare.10 (modify this as Ayushman Bharat since 2018 is constituted to help these people and you have also taken up such people)

We have deleted this line and merged this paragraph with the previous one as follows:

“**After the outbreak of COVID-19 in India, the Government of India imposed a nationwide lockdown from 25 March, 2020 to 31 May, 2020,^9^ and subsequently announced various pandemic relief measures for the poor.^10^ However, despite the increased disease risk, most pandemic relief measures announced by the central and state governments of India do not make provisions for PEHs.^11^ This may be due to the paucity of research focusing on the unique challenges faced by PEHs during the pandemic**.” (Lines 54–61).

Reference 10 has been changed to: **Reuters. India unveils $23bn package to help poor hit by COVID-19 lockdown. Al Jazeera. Published May 26, 2020. Accessed December 19, 2020. https://www.aljazeera.com/economy/2020/3/26/india-unveils-23bn-package-to-help-poor-hit-by-covid-19-lockdown**

Line 89

purposive homogeneous sampling, (what was homogenous here) , you have gender stratification , so lets put it as purposive sampling

We changed **purposive homogeneous sampling** to **purposive sampling** (Line 91).

We also changed Line 137 – 138 from “**The homogeneity of the participants can be seen from Table 1**.” To “**The participant characteristics are presented in Table 1**.”

line 666

one cannot anticipate no difference, not taking those without AC can be stated as a frank limitation

We stated the non-recruitment of PEHs without Ayushman Bharat cards as a frank limitation by changing Lines 662–671 from **“Additionally, as the organization that facilitated this study ensured that all PEHs who engaged with it accessed all government schemes available to them, we could not recruit a PEH who did not have an Ayushman Bharat card in this study. The lived experiences of PEHs who do not have their Ayushman Bharat cards may be different to those who have their cards due to a potential difference in their perceived access to healthcare; however, we do not anticipate this difference to be significant as the participants in this study expressed their negative experiences with using the private hospitals—where the Ayushman Bharat card is required—and their subsequent decision to only use the public hospitals, where treatment is free and the card is not needed, thereby negating their advantage of having the card.”** To **“Additionally, as the organization that facilitated this study ensured that all PEHs who engaged with it accessed all government schemes available to them, we could not recruit PEHs who did not have an Ayushman Bharat card in this study—the lived experiences of PEHs who do not have their Ayushman Bharat cards may be different to those who have their Ayushman Bharat cards due to a potential difference in their perceived access to healthcare.”**

Journal Requirements:

Please review your reference list to ensure that it is complete and correct. If you have cited papers that have been retracted, please include the rationale for doing so in the manuscript text, or remove these references and replace them with relevant current references. Any changes to the reference list should be mentioned in the rebuttal letter that accompanies your revised manuscript. If you need to cite a retracted article, indicate the article’s retracted status in the References list and also include a citation and full reference for the retraction notice.

We confirm that the reference list is complete and correct and we have not cited any retracted papers. We changed reference 10 because we deleted a sentence that was supported by reference 10 and added a new sentence supported by a new reference in its place.

Old reference 10: **Baseline Study- “Enabling Inclusive Cities for Homeless.” Indo-Global Social Service Society & Organisation Functioning for Eytham’s. Published May 1, 2019. Accessed July 11, 2021.** [**https://igsss.org/wp-content/uploads/2019/10/Baseline-Study-Enabling-Inclusive-Cities-for-Homeless.pdf**](https://igsss.org/wp-content/uploads/2019/10/Baseline-Study-Enabling-Inclusive-Cities-for-Homeless.pdf)

New reference 10: **Reuters. India unveils $23bn package to help poor hit by COVID-19 lockdown. Al Jazeera. Published May 26, 2020. Accessed December 19, 2020. https://www.aljazeera.com/economy/2020/3/26/india-unveils-23bn-package-to-help-poor-hit-by-covid-19-lockdown**
