# Supplementary material for: Experiencing the COVID-19 pandemic as a homeless person in Chennai, India: An interpretative phenomenological analysis
Source: PLoS One. 2023 Nov 30;18(11):e0295164. doi: 10.1371/journal.pone.0295164 (PMC10688851; doi:10.1371/journal.pone.0295164)
Supplement: Attachment — Submitted filename: Response-to-reviewers-3.2.docx [file pone.0295164.s006.docx]

**RESPONSE TO REVIEWERS**

Reviewer #1: Please note that I’m invited to review the second revision of the manuscript. I have not reviewed previous versions of this manuscript.
The authors have performed an important study about homelessness in India during the COVID-19 pandemic. There were nine participants which were carefully approached to participate in the study. The authors performed semi-structured interviews and analyzed data with an interpretative phenomenological analysis.

**Response**: We wish to thank the reviewer for the meticulous review and the largely positive assessment of our work. We have provided a point-by-point response to the comments raised in the table below.

| **Section** | **Reviewer’s comments** | **Authors’ response** | **Changes** |
| --- | --- | --- | --- |
| **Abstract** | The authors should state that they performed a qualitative study (methods) | We have added that we conducted a qualitative study in the abstract. | To explore the lived experiences of PEHs during the COVID-19 pandemic, a qualitative study—interpretative phenomenological analysis—involving semi-structured interviews of nine PEHs was conducted in Chennai, India, at food stalls frequented by them, between 14–25 September, 2020. |
|  | And might describe some participant characteristics (results) in the Abstract. | We have added a line on participant characteristics in the abstract. | All the participants were migrant workers living alone, and were the sole breadwinners of their families. |
|  | The results are well-presented, however, they should be shortened to meet the word limit. | We have ensured that the abstract is within the 300-word limit prescribed by PLOS ONE. | ---- |
|  | The implications should be written out rather than enumerated. | We have written out the implications instead of enumerating them in the abstract. | The study highlights several important policy implications. Firstly, it underscores the importance of involving social workers to facilitate communication between healthcare providers and patients from vulnerable communities. This engagement can help minimize discrimination and promote equitable access to healthcare. Secondly, the study emphasizes the need for effective public health communication. Specifically, there is a need to address and alleviate concerns about the transmission of COVID-19 within hospital premises. Lastly, the research suggests that government initiatives aimed at fostering community participation should persist both during and after the pandemic. |
| **Introduction** | The introduction starts with the main topic ‘homelessness’. The authors succeed in briefly summarizing and clearly presenting the need for the study. The introduction section does not require revisions in my opinion. | We thank the reviewer for the positive appraisal of the introduction section. | ---- |
| **Methods** | The methods section should start with an introduction of ‘phenomenology’ (design) rather than ‘interpretative phenomenological analysis’ (data-analysis). | We have added a line to define phenomenology at the beginning of the methods section. | Phenomenology is a philosophical approach to the study of experience, which seeks to understand and describe lived experiences. |
|  | Why did the authors chose to explore the phenomenon from an interpretative phenomenological perspective? | We have added the reason for choosing the IPA method in the first paragraph of the methods section. | Among the various approaches to qualitative research—such as phenomenology, grounded theory, discourse analysis, and narrative analysis—the phenomenological method was selected because the study aims to comprehend the lived experience of a particular phenomenon (enduring the COVID-19 pandemic and the resultant lockdown) by a specific group of people (PEHs). As understanding the lived experience involves an in-depth investigation into meaning-making, hermeneutic, existential, or interpretative phenomenology were considered appropriate. Among these approaches, interpretative phenomenological analysis (IPA) was adopted because this method analyzes the convergence and divergence of individual participants' experiences while simultaneously retaining their individuality. |
|  | The participants and setting sections are well-written. I do have two questions about the prompting. Is it possible to present all prompting questions in a box/table? And why did the authors not include questions about homelessness in the prompting? It now looks like that COVID-19 is the topic of the study instead of homelessness. | We defined the phenomenon of interest as the lived experience of the COVID-19 pandemic, and the population of interest as individuals experiencing homelessness in Chennai, India; therefore, we did not add any questions about homelessness as it was not the main focus of the study. We have clarified the phenomenon and population of interest in the methods section.  We have provided all the prompting questions in the supplementary material (S2: Prompting Questions). This material is cited in the Setting and data acquisition subsection.  We thank you for positively judging the participants and setting subsections. | …the study aims to comprehend the lived experience of a particular phenomenon (enduring the COVID-19 pandemic and the resultant lockdown) by a specific group of people (PEHs). |
|  | I do miss a ‘trustworthiness’ paragraph summarizing the most important techniques to increase trustworthiness of results. It may be helpful to structure the Methods section according to the Standard for Reporting Qualitative Research reporting guideline (SRQR). | We have amended the Ethical considerations section to Ethical considerations and trustworthiness section and summarized the techniques employed to increase the trustworthiness of the results. We can confirm that all the information required by the SRQR guideline have been provided in the manuscript. | **Ethical considerations and trustworthiness**  The study was conducted in accordance with the national guidelines and the Declaration of Helsinki. All procedures were approved by Sri Jagannath Healthcare and Research Center – Independent Ethics Committee, Dhanbad, India (No: SCR013/2020). This study ensures trustworthiness by employing several strategies. Credibility is upheld through prolonged engagement with participants, while transferability is facilitated by providing context-rich descriptions. Dependability is maintained through comprehensive documentation of the research process, and confirmability is achieved via peer debriefing, enhancing the overall validity and rigor of the findings. |
| **Results** | The results section needs no major changes in my opinion. The themes are illustrative and the stories are breath-taking. I would, however, like the authors to bring the terms such as 'individual themes' and 'master themes' in line with IPA according to Smith. Terms used by Smith et al are, amongst others, notes, emerging themes (may match individual theme), and patterns (may match master theme?). The case descriptions are very informative and suit a description of IPA-results. | We thank the reviewer for the positive appraisal of the results section. We have updated our terminology according to the latest book by Smith and Nizza (Smith, J. A., & Nizza, I. E. (2022). *Essentials of interpretative phenomenological analysis.* American Psychological Association. [https://doi.org/10.1037/0000259-000](https://psycnet.apa.org/doi/10.1037/0000259-000)) as follows:  Individual themes –> personal experiential themes  Master themes –> group experiential themes  Emergent theme –> experiential statement  Subtheme remains the same | ---- |
| **Discussion** | The discussion section needs to be shortened. Please discuss 2, max. 3 topics instead of the current 6. | We understand your concern about the length of the Discussion section and the suggestion to discuss a smaller number of topics. We would like to explain our rationale for the current length of the Discussion.  Given the unique nature of our study's focus on homeless migrant workers in Chennai during the COVID-19 pandemic, we believe that a comprehensive discussion is crucial to provide a thorough understanding of their experiences. This is a vulnerable population and research on homeless individuals in India is limited. Our goal was to shed light on various aspects of their lived experiences, which we believe is valuable in drawing the attention of policymakers, who have not as yet made any provisions for the rehabilitation of this population post the lifting of lockdown.  While we acknowledge that discussing fewer topics might streamline the Discussion section, we also believe that a nuanced exploration of multiple facets of our participants' experiences is vital in order to capture the complexity of their situations and thereby, provide insights that can inform policy decisions related to vulnerable populations.  We would be happy to consider any specific recommendations you might have for condensing the Discussion section without compromising the depth of our analysis.  Please note that the discussion section was 1576 words in the previous submission. We have brought it down to 1390 words in this submission by using more concise language. | ---- |
|  | Please provide ‘strenghts’ of the study before ‘limitations’. Maybe shorten the limitations to max. 8 lines. | We have added a paragraph on the strengths before the discussion of the limitations. We have shortened the limitations to 8 lines. | The strength of our study lies in its thorough and comprehensive exploration of participants' lived experiences. Given the heterogeneity of our participants, our study offers a diversity of perspectives that enriches our analysis, providing depth and complexity to our understanding of the phenomenon under investigation. Our study holds significance in contributing to the broader understanding of the interplay among socioeconomic factors, public health, and the process of meaning-making.  A key limitation of our study is that the experiences of PEHs in other locations may differ from those in Chennai. Therefore, our findings cannot be generalized. Additionally, as Samanjasa Foundation ensured that PEHs who engaged with it accessed all government schemes available to them, we could not recruit PEHs without Ayushman Bharat cards—their lived experiences may differ from those with Ayushman Bharat cards due to a potential difference in their perceived access to healthcare. Thus, our findings cannot be generalized. Furthermore, active government and NGO involvement during the lockdown may have influenced our results. To comprehend the true aftermath, research when conditions return to pre-pandemic norms is essential. |
| **Conclusions** | In the conclusion, more use can be made of the patterns found in the study data. | We have added a summary of the key findings to the conclusions section. | **Conclusions**  The present study has uncovered significant patterns within the lived experiences of PEHs in Chennai during the COVID-19 pandemic. These patterns, encompassing various thematic dimensions, highlight the multifaceted impact of the pandemic on this vulnerable population. The participants' attitudes towards COVID-19, initially characterized by denial and lack of fear, evolved into complex emotions including negative perceptions towards the rich, risk acceptance, and a palpable fear. The pandemic's broader implications on their lives were manifested through isolation, loss of livelihood, challenges in building trust within work environments, and unexpected positive experiences amid adversity. The study's exploration of the pandemic's impact on mental health unveiled a range of responses, including heightened anxiety, loss of hope, interpersonal stress, uncertainty, and expressions of anger towards socio-economic disparities. Notably, the coping strategies employed by the participants spanned social media engagement, participation in collective activities, religious practices, and substance use. Furthermore, the study illuminated the barriers and facilitators that shaped healthcare access in PEHs. While reluctance to use both public and private hospitals was observed, the provision of free testing and quarantine facilities emerged as significant facilitators in this context.  With the COVID-19 pandemic continuing to spread in India, and the state and central governments still not promulgating COVID-19-related policies directed at PEHs, they continue to suffer through the pandemic, without adequate access to services. Ensuring safe accommodation can improve the physical and mental resilience of PEHs against COVID-19; we hope our findings trigger a policy dialogue to provide them directed COVID-19 relief. |
| **Language and formatting** | The manuscript is well formatted. | Thank you. | ---- |
|  | English language use can be a bit more academic here and there. | We have gone through the manuscript and replaced any informal words with formal equivalents wherever possible. |  |
|  | I also recommend formatting the tables in APA style. | We have formatted the tables in the APA style. |  |
|  | I recommend minor revisions. | We hope our revisions are to your satisfaction. |  |

Journal Requirements:
Please review your reference list to ensure that it is complete and correct. If you have cited papers that have been retracted, please include the rationale for doing so in the manuscript text, or remove these references and replace them with relevant current references. Any changes to the reference list should be mentioned in the rebuttal letter that accompanies your revised manuscript. If you need to cite a retracted article, indicate the article’s retracted status in the References list and also include a citation and full reference for the retraction notice.

**Response**: Our reference list is complete and correct. No retracted papers have been cited. There are no changes to the reference list.
