# Supplementary material for: Experiencing the COVID-19 pandemic as a homeless person in Chennai, India: An interpretative phenomenological analysis
Source: PLoS One. 2023 Nov 30;18(11):e0295164. doi: 10.1371/journal.pone.0295164 (PMC10688851; doi:10.1371/journal.pone.0295164)
Supplement: Attachment — Submitted filename: Response-to-reviewers-3.3.docx [file pone.0295164.s007.docx]

**RESPONSE TO REVIEWERS**

**Journal Requirements:**

Please review your reference list to ensure that it is complete and correct. If you have cited papers that have been retracted, please include the rationale for doing so in the manuscript text, or remove these references and replace them with relevant current references. Any changes to the reference list should be mentioned in the rebuttal letter that accompanies your revised manuscript. If you need to cite a retracted article, indicate the article’s retracted status in the References list and also include a citation and full reference for the retraction notice.

**Response**: Our reference list is complete and correct. No retracted papers have been cited. There are no changes to the reference list.

**Reviewers' comments:**

Comments to the Author

1. If the authors have adequately addressed your comments raised in a previous round of review and you feel that this manuscript is now acceptable for publication, you may indicate that here to bypass the “Comments to the Author” section, enter your conflict of interest statement in the “Confidential to Editor” section, and submit your "Accept" recommendation.

Reviewer #1: All comments have been addressed

**Response:** Thank you for your positive appraisal.

2. Is the manuscript technically sound, and do the data support the conclusions?

The manuscript must describe a technically sound piece of scientific research with data that supports the conclusions. Experiments must have been conducted rigorously, with appropriate controls, replication, and sample sizes. The conclusions must be drawn appropriately based on the data presented.

Reviewer #1: Yes

**Response:** Thank you for your positive appraisal.

3. Has the statistical analysis been performed appropriately and rigorously?

Reviewer #1: N/A

**Response:** N/A

4. Have the authors made all data underlying the findings in their manuscript fully available?

The PLOS Data policy requires authors to make all data underlying the findings described in their manuscript fully available without restriction, with rare exception (please refer to the Data Availability Statement in the manuscript PDF file). The data should be provided as part of the manuscript or its supporting information, or deposited to a public repository. For example, in addition to summary statistics, the data points behind means, medians and variance measures should be available. If there are restrictions on publicly sharing data—e.g. participant privacy or use of data from a third party—those must be specified.

Reviewer #1: No

**Response:** The data cannot be made publicly available due to privacy concerns. The data has been deposited with the ethics committee as per the data policy and upon reasonable request, the secretary of the ethics committee will make the raw data available. As per the data policy of PLOS ONE, the contact details of the secretary have been uploaded on the submission system, in the data availability statement.

5. Is the manuscript presented in an intelligible fashion and written in standard English?

PLOS ONE does not copyedit accepted manuscripts, so the language in submitted articles must be clear, correct, and unambiguous. Any typographical or grammatical errors should be corrected at revision, so please note any specific errors here.

Reviewer #1: Yes

**Response:** Thank you for your positive appraisal.

6. Review Comments to the Author

Please use the space provided to explain your answers to the questions above. You may also include additional comments for the author, including concerns about dual publication, research ethics, or publication ethics. (Please upload your review as an attachment if it exceeds 20,000 characters)

Reviewer #1: First of all, I would like to thank the authors for the excellent response to reviewer(s). It was pleasant to read and finely structured. In addition, the points of interest were satisfactorily incorporated into the manuscript. Below are just a few minor comments worth processing.

**Response:** Thank you for your positive appraisal. We have endeavoured to respond to all your comments below. We would like to express our sincere gratitude to you for your effort in improving our manuscript, which upon publication, will serve as the basis for our white paper to the government regarding provision of services to persons experiencing homelessness. We sincerely hope that our paper will have real-world implications for persons experiencing homelessness in India, and we thank you in helping us make our paper as impactful as it can be so that we can achieve our objective. Below are our responses to your comments on each section.

| **Section** | **Reviewer’s comments** | **Authors’ response** | **Changes** |
| --- | --- | --- | --- |
| **Abstract** | The abstract now contains all relevant information. Spelling and grammar needs a final check. E.g., the sentence "To explore the lived experiences of PEHs during the COVID-19 pandemic, a qualitative study-interpretative phenomenological analysis-involving semi-structured interviews of nine PEHs was conducted in Chennai, India, at food stalls frequented by them, between September 14-25, 2020." does not read pleasantly and seems grammatically incorrect. Perhaps the text could be modified to "We conducted a qualitative study to investigate the lived experiences of PEHs during the COVID-19 pandemic. Data were collected with semi-structured interviews in nine PEHs from Chennai, India, recruited at food stalls between Septembre 14-25, 2020. We analyzed the data with interpretive phenomenological analysis". | We have made the change suggested by you. This increased the word count beyond the 300-word limit. So, we edited the sentences for brevity. We also made additional changes suggested by you regarding using “we” instead of “the study”; this reduced the word count to within 300 words. We also proofread the entire manuscript and ensured there are no long winding sentences. | Accordingly, we conducted a qualitative study on their lived experiences during the COVID-19 pandemic. Semi-structured interviews were conducted in nine PEHs from Chennai, India, recruited at food stalls between September 14–25, 2020. Data were analyzed using interpretive phenomenological analysis. |
| **Introduction** | No remarks. | Thank you for taking the time to apprise this section. | ---- |
| **Methods** | The method section has improved tremendously. Certain choices, such as the choice of phenomenology, are now clearly justified. The credibility of the study is now easier to assess. Thanks for the modifications. | We thank the reviewer for the positive appraisal of the methods section. | ---- |
| **Results** | The terminology (e.g., personal experiential themes) is now uniform and easier to understand. | We thank the reviewer for the positive appraisal of the results section. | ---- |
| **Discussion** | The arguments in lines 640-647 are interesting but speculative in nature. In my opinion, this text can be removed. | As the argument in these lines were presenting results that were not presented in the results section, it is understandable that they were apprised as speculative. We have moved these lines to the results section, under “impact of COVID-19 on mental health” and wrote a line to provide context on why a psychiatrist evaluated the participants. We then wrote a brief sentence discussing this result in the discussion section. | In results (impact of COVID-19 on mental health):  As the participants exhibited significant anxiety and depression symptoms, they were referred to an independent psychiatrist as mandated by the risk reduction policies of the Sri Jagannath Healthcare and Research Center – Independent Ethics Committee, Dhanbad, India. Upon evaluation and follow-up, the independent psychiatrist found that the participants did not meet the DSM-V diagnostic criteria for generalized anxiety disorder or major depression, and were determined to be at minimal risk for suicidality.  In discussion:  The participants presented minimal suicide risk, suggesting that they had evolved robust coping mechanisms. |
|  | The sentence "The pandemic exacerbated substance use, as reported by studies conducted globally." seems redundant and insufficiently substantiated. Please remove. | We apologise that we simply wrote the sentence without expanding on why we included it in the discussion. We have added a sentence on why this sentence was included in the discussion. If the reviewer feels that we must still remove the sentence, kindly let us know and we will remove it. | Similar to a previous study,^22^ the pandemic exacerbated substance use in our participants. The government must accordingly create effective public health messages targeting drug use among PEHs and increase the capacity of its drug deaddiction program. |
| **Conclusions** | No remarks. The patterns are now well explained and summarized in a concise conclusion. | We thank the reviewer for the positive appraisal of the conclusions section. | ---- |
| **Language** | More use could be made of 'we'-perspective instead of "the study" or some other third-person perspective. The researchers participate in the study as interviewers. Therefore, it seems appropriate to change, for example, "The study's exploration of the pandemic's impact" to "We explored the pandamic's impact." Please check the manuscript for use of 'we'-perspective where possible. | We have gone through the entire manuscript and replaced “the study” with “we” perspective wherever possible. We also edited the sentences to correct any grammatical errors arising from such changes. | ---- |

Apart from the above changes, the journal had requested that we remove any funding information from the manuscript. We have therefore, removed all references to a “sponsoring agency” and the study being “commissioned” from the manuscript. The following are the changes:

**The study was conducted with the help of the Savitri Ghantasala Center for Health Equity, Samanjasa Foundation, Chennai, India—a non-governmental organization (NGO) that engages with PEHs in Chennai and provides them various services including emergency interest-free loans, hot meals, medical checkups, and facilitating access to government schemes and facilities. They continued providing these services during the COVID-19-induced lockdown. Their case workers disseminated information about the study to PEHs who accessed their services. To build rapport with PEHs, a case worker arranged for the authors to meet the PEHs who had expressed interest in knowing more about the study at a location they are familiar with—the food stalls frequented by them—on two occasions. At these icebreaker sessions, the authors shared a meal with PEHs and engaged in casual conversation.**
